# A hypomorphic allele of *SLC35D1* results in Schneckenbecken-like dysplasia

**DOI:** 10.1093/hmg/ddz200

**Published:** 2019-08-19

**Authors:** Carsten Rautengarten, Oliver W Quarrell, Karen Stals, Richard C Caswell, Elisa De Franco, Emma Baple, Nadia Burgess, Roobin Jokhi, Joshua L Heazlewood, Amaka C Offiah, Berit Ebert, Sian Ellard

**Affiliations:** 1 School of BioSciences, The University of Melbourne, Victoria 3010, Australia; 2 Department of Clinical Genetics, Sheffield Children’s Hospital, Western Bank, Sheffield S10 2TH, UK; 3 Department of Molecular Genetics, Royal Devon & Exeter NHS Foundation Trust, Barrack Road, Exeter, EX2 5DW, UK; 4 University of Exeter School of Medicine, Barrack Road, Exeter EX2 5DW, UK; 5 Department of Histology, Sheffield Children’s Hospital NHS Foundation Trust, Western Bank, Sheffield UK. S10 2TH, UK; 6 Department of Obstetrics and Gynaecology, Sheffield Teaching Hospitals, Jessop Wing Tree Root Walk, Sheffield S10 2SF, UK; 7 University of Sheffield, Academic Unit of Child Health, Sheffield Children’s Hospital NHS Foundation Trust, Western Bank, Sheffield S10 2TH, UK

**Keywords:** Schneckenbecken dysplasia, SLC35D1, nucleotide sugar transporters, genetics, radiology

## Abstract

We report the case of a consanguineous couple who lost four pregnancies associated with skeletal dysplasia. Radiological examination of one fetus was inconclusive. Parental exome sequencing showed that both parents were heterozygous for a novel missense variant, p.(Pro133Leu), in the *SLC35D1* gene encoding a nucleotide sugar transporter. The affected fetus was homozygous for the variant. The radiological features were reviewed, and being similar, but atypical, the phenotype was classified as a ‘Schneckenbecken-like dysplasia.’ The effect of the missense change was assessed using protein modelling techniques and indicated alterations in the mouth of the solute channel. A detailed biochemical investigation of SLC35D1 transport function and that of the missense variant p.(Pro133Leu) revealed that SLC35D1 acts as a general UDP-sugar transporter and that the p.(Pro133Leu) mutation resulted in a significant decrease in transport activity. The reduced transport activity observed for p.(Pro133Leu) was contrasted with *in vitro* activity for SLC35D1 p.(Thr65Pro), the loss-of-function mutation was associated with Schneckenbecken dysplasia. The functional classification of SLC35D1 as a general nucleotide sugar transporter of the endoplasmic reticulum suggests an expanded role for this transporter beyond chondroitin sulfate biosynthesis to a variety of important glycosylation reactions occurring in the endoplasmic reticulum.

## Introduction

Schneckenbecken dysplasia is a rare recessive lethal chondrodysplastic condition which has the classical radiological signs of rounded ilia and medial protrusions of horizontally orientated acetabula, reminiscent of a snail-like configuration, hence the name (1–3). Furthermore, it is associated with fetal hydrops (3). Two loci have been implicated in Schneckenbecken dysplasia, *SLC35D1*, which encodes a nucleotide sugar transporter (NST) (4), and *INPPL1* encoding a inositol polyphosphate phosphatase-like type protein (5).

In humans, NSTs belong to the solute carrier (SLC) group 35 (SLC35), a family that currently contains a total of 31 members (6). SLC35D1 was first identified as hUGTrel7, a transporter located in the endoplasmic reticulum (ER) in mammalian cells (7). A yeast heterologous expression assay demonstrated that SLC35D1 could initially facilitate the transport of UDP-glucuronic acid (UDP-GlcA) and UDP-*N*-acetylgalactosamine (UDP-GalNAc) (7) and later also UDP-*N*-acetylglucosamine (UDP-GlcNAc) (4). However, a number of other UDP-sugars could not be conclusively excluded from the initial study, including UDP-galactose (7). Since both, UDP-GlcA and UDP-GalNAc, are substrates for chondroitin sulfate and dermatan sulfate biosynthesis, SLC35D1 has been directly associated with a role in chondroitin sulfate biosynthesis (4). Chondroitin sulfate chains contain repeating disaccharide units of GlcA and GalNAc, with the latter often harbouring sulfates at either the C6 or C4 position. These repeats are the primary components of glycosaminoglycan (GAG) chains found in cartilage proteoglycans (8), and their length and content are essential for cartilage development, specifically in the formation of epiphyseal cartilage. The significance of SCL35D1 involvement in chondroitin biosynthesis has been confirmed through the analysis of SLC35D1-deficient mice. Knockout mice developed a lethal form of skeletal dysplasia with severe defects in cartilage and skeleton formation that were caused by defects in chondroitin sulfate biosynthesis as indicated by the production of short and sparse chondroitin sulfate chains (4).

Prenatal detection of a skeletal dysplasia poses a significant genetic counselling challenge because of the heterogeneous genetic aetiology of these conditions. Here we describe a case of a family who lost a total of six pregnancies, four of which were associated with multiple congenital anomalies. Post mortem and radiological examination following the last pregnancy loss did not reveal a specific diagnosis. Parental exome sequencing identified a novel variant of the *SLC35D1* gene, suggesting a diagnosis of Schneckenbecken dysplasia (4, 9). In silico protein modelling and a detailed biochemical characterisation confirmed that the new SLC35D1 variant is functionally compromised.

## Results

### Phenotype, pathology and radiology

**Figure 1:**
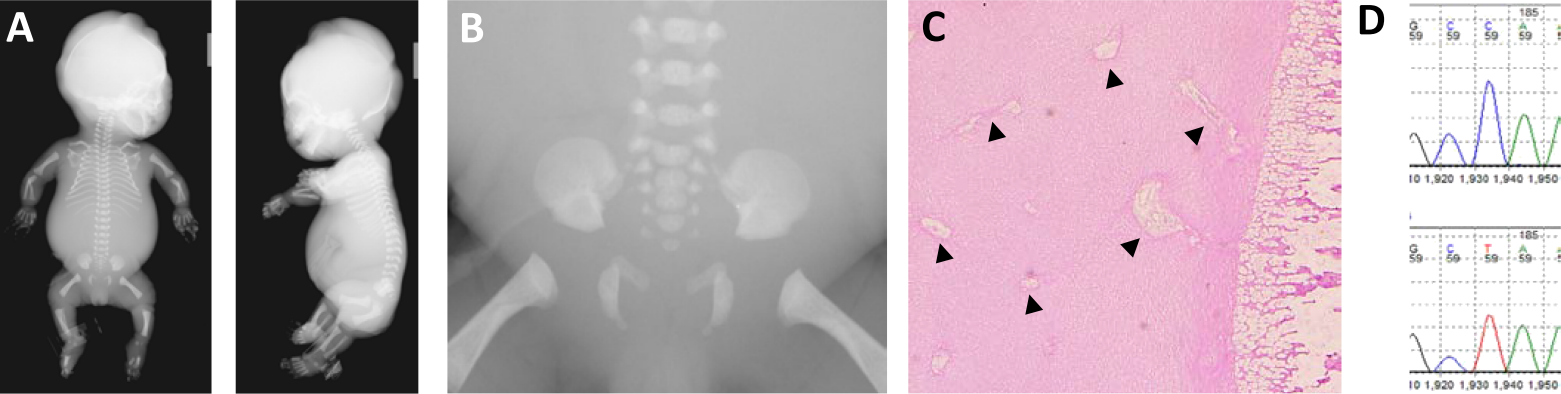
Phenotype and genotype of the affected fetus (MCA 4) (A) Radiographs of the proband (MCA 4). (B) Radiograph outlining the absence of a snail-like ilia in the proband (MCA 4). (C) Epiphyseal plate of femur, featuring an increase in vascular channels within the resting cartilage (indicated by arrows). (D) Sanger sequencing of the homozygous c.398C > T variant in the affected fetus.

A consanguineous Arabian couple had eight pregnancies with two live born children and an early miscarriage. The first pregnancy was initially uneventful but ended with a fetal death at 21 weeks. A post-mortem examination showed no malformations and no radiological evidence of a skeletal dysplasia. The cause of death was attributed to early placental separation. Brief clinical details of the four pregnancies affected by multiple congenital anomalies are summarised in Table 1. A post-mortem examination was only available for the fourth pregnancy, MCA 4 in Table 1 (Fig. 1A; 1B). Examination showed extreme shortening and bowing of the long bones; a small, narrow thorax and prominent abdomen; a disproportionately large head; a small mouth with a cleft soft palate; large ears with unfolded helices; hirsutism, overlapping fingers and clinodactyly; bilateral rocker bottom heels; kyphoscoliosis; mild bilateral hydronephrosis and pulmonary hypoplasia. There was no cardiac anomaly. Resting cartilage of the femur showed an increase in vascular channels with mild perivascular fibrosis (Fig. 1C). The findings on anteroposterior and lateral radiographs of the fetus are summarised in Supplementary Table 2 and are compared with the radiological features previously reported for Schneckenbecken dysplasia (9). The radiological features in this case are milder than usually seen, and the absence of a snail-like pelvis meant that Schneckenbecken dysplasia was not considered within the differential diagnosis, with the primary suggested diagnosis being a type II collagenopathy (although the normal ossification of the superior pubic rami was noted).

**Table 1:** Summary of pregnancies affected by multiple congenital anomalies (MCA)

**Pregnancy Number**	**Cystic hygroma/nuchal translucency**	**Ascites/hydrops**	**Short long bones**	**Cleft Palate**	**Cardiac defect †**	**outcome**
MCA 1	+	+	+	ND	+	IUFD at 21 weeks
MCA 2	+	ND	+	ND	ND	TOP 12 weeks
MCA 3	+	+	+	ND	ND	TOP 15 weeks
MCA 4	+	+	+	+ (soft)	−	IUFD at 28 weeks

† Ventricular septal defect with possible aortic stenosis and coarctation or hypoplasia of the aortic arch.

IUFD = intra-uterine fetal death; TOP = termination of pregnancy

### Sequence analysis of parents

To identify variants that could be attributed to these congenital anomalies, parental exome sequencing was performed. Sequencing determined that the parents were both heterozygous for a missense change in the solute carrier *SLC35D1* (NM_015139.3) c.398C > T p.(Pro133Leu) (Fig S1). This variant is not present in the Genome Aggregation Database (gnomAD) database of > 120 000 individuals (10) and affects a highly conserved amino acid. The *in silico* pathogenicity prediction tools Align GVGD (11, 12), SIFT (J. Craig Venter Institute; La Jolla, CA; http://provean.jcvi.org/index.php) and PolyPhen (13) all predicted the variant to have a detrimental effect on protein function; specific grades or scores returned by the prediction tools were: Align GVGD, class C65 (probability for pathogenicity ≥0.81) (14); SIFT, 0.00 (SIFT scores of ≤ 0.05 are usually taken as indicative of deleterious substitutions) (15); PolyPhen HumDiv, score (probability for pathogenicity) 0.998, sensitivity 0.27, specificity 0.99; PolyPhen HumVar, score 0.958, sensitivity 0.63, specificity 0.92. Sanger sequencing of the DNA sample from one of the affected fetuses identified the *SLC35D1* missense variant, p.(Pro133Leu), in the homozygous state, consistent with autosomal recessive inheritance (Fig. 1D).

### Structural modelling of SLC35D1 and the p.(Pro133Leu) variant

**Figure 2:**
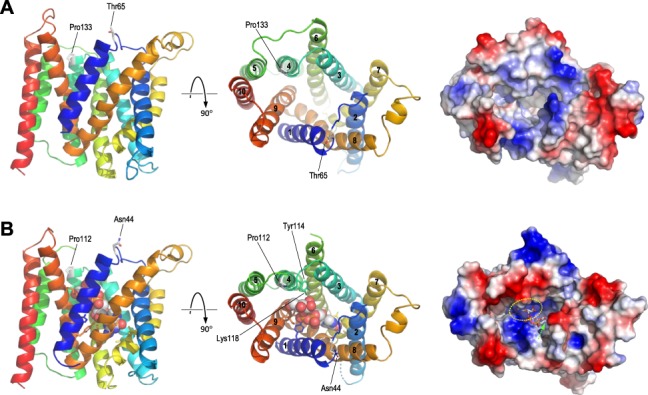
Predicted structure of SLC35D1. (A) Predicted structure of SLC35D1, residues 44–338, modelled on PDB 5ogk (yeast GDP-mannose transporter Vrg4). Left and centre panels show the protein in ribbon format, coloured from N-terminal, blue, toC-terminal, red, with sidechains shown in stick format for residues Pro133 and Thr65; views shown are: left, in the plane of the ER membrane with the lumenal surface uppermost; and centre, from the ER lumen, with TM helices numbered 1–10. The right panel shows the predicted molecular surface, seen from the lumen, coloured by surface charge (blue, positive/basic; red, negative/acidic). (B) as A, but showing the structure of Vrg4 bound to GDP-mannose (PDB 5ogk); in left and centre panels, Pro112 and Asn44, the equivalent residues to SLC35D Pro133 and Thr65, are shown in stick format and coloured by atom type; sidechains, coloured by protein position, are also shown for residues which contribute directly to substrate binding in the nucleobase- or sugar-binding pockets, with those in TM helix 4 (Tyr114, Lys118) labelled. The GDP-mannose substrate is shown by space-filling spheres in the left and centre panels, and in stick format in the right panel. The yellow oval shows the approximate location of the sugar-binding pocket, while the nucleobase-binding pocket is buried deeper within the channel as indicated by the green arrow.

While there are no experimental structures for the SLC35D1 protein, structures have been reported for a number of other NSTs allowing comparative protein modelling. A search for suitable templates using the Swiss-Model web server (16) identified structures of the yeast GDP-mannose transporter Vrg4 (17) as the best available templates, with overall 41.4% sequence similarity and 20.4% identity with SLC35D1. Modelling on the nucleotide sugar-bound form of Vrg4 (PDB identifier 5ogk) yielded a structure covering residues 44 to 338 of SLC35D1; a model constructed on the apo-form of Vrg4 (PDB 5oge) was essentially identical (root-mean-square deviation between α-carbons = 0.65 Å; data not shown). Consistent with the known structure of Vrg4 and other NSTs as integral membrane proteins, the predicted structure was of a roughly barrel-shaped protein containing the characteristic 10 transmembrane (TM) helices surrounding a central channel (Fig. 2A). In the modelled structure, the transporter is open to the lumen of the ER, with both Pro133 and Thr65 lying roughly opposite each other at the periphery of the substrate channel on this surface. In keeping with the different substrate specificities of SLC35D1 and Vrg4, the predicted protein surface showed differences in topology and, more notably, surface charge within the substrate channel (Fig. 2, right panels). These differences were also evident in the aligned sequences of the two proteins. In Vrg4, a conserved FYNN motif (residues 218–221) in the nucleobase binding pocket of the channel confers specificity for guanine-containing nucleotide sugars, while the GALNK motif in the sugar-binding pocket (residues 285–289) is the consensus sequence observed in NSTs for which mannose is the preferred sugar (17). In the modelled SLC35D1 structure, these motifs are replaced by YYNA (residues 239–242) and GCIKN (289–293), respectively, consistent with the different specificities of SLC35D1 for both nucleobase and sugar groups of the substrate. Use of other NST structures as modelling templates (specifically, PDB identifiers 6i1z and 6i1r, the apo- and CMP-bound forms respectively of maize CMP-sialic acid transporter; and PDB 6oh2, CMP-bound form of mouse CMP-sialic acid transporter) yielded models of essentially identical structure and topology to those obtained for the Vrg4 structures (data not shown).

**Figure 3:**
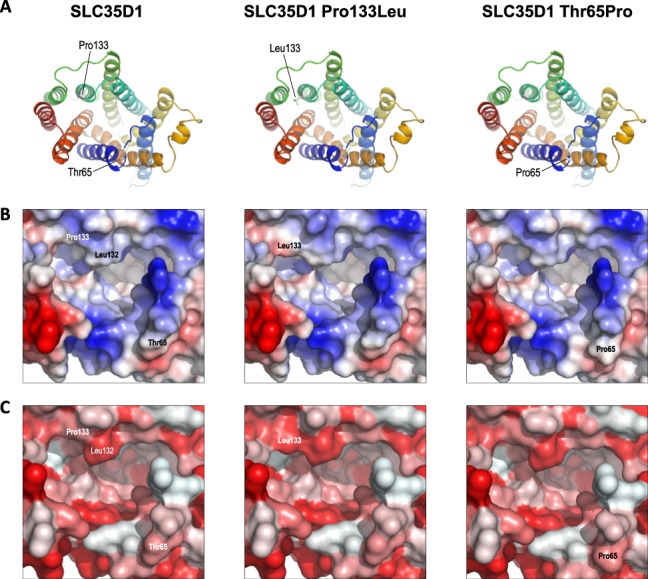
Predicted structure of SLC35D1 variants. (A) The left panel shows the predicted structure of SLC35D1 as seen from the ER lumen, as in Fig. 2A; centre and right panels show the predicted structures of the p.(Pro133Leu) and p.(Thr65Pro) variants respectively, derived from *in silico* mutagenesis of the SLC35D1 structure. (B) Predicted surface charge (blue, positive/basic; red, negative/acidic) around the substrate channel at the lumenal surface in SLC35D1 (left) and the p.(Pro133Leu) and p.(Thr65Pro) variants (centre and right respectively); labels indicate surface positions of relevant residues. C) as B, but coloured by predicted surface hydrophobicity (red, most hydrophobic, to white, most polar).

Detailed inspection of the predicted structures showed that Pro133 lies at the N-terminal of TM helix 4, separating this helix from the preceding helix 3–helix 4 loop, while Thr65 lies in the flexible helix 1–helix 2 loop. Introduction of the p.(Pro133Leu) and p.(Thr65Pro) variants into the model showed that both novel amino acids could be accommodated without significant perturbation of the overall structure (Fig. 3A), although both variants, and p.(Pro133Leu) in particular, were predicted to cause modest changes to the surface properties of the protein around the substrate channel (Fig. 3B, C). It is possible that such changes might result in altered binding or release of the transported substrate into the ER lumen, particularly as Pro133 lies just N-terminal to Phe135 and Arg139 in helix 4 which, by homology with Vrg4 Tyr114 and Lys118, are predicted to contribute to substrate binding in the sugar-binding pocket (17); moreover, Pro133 lies immediately adjacent to Leu132, which appears to partially cap the sugar-binding pocket at the luminal surface. Notwithstanding these potential effects, it is also possible that both variants have an impact upon protein structure and function which is not evident from comparative modelling alone. The technique of comparative modelling is limited in that it relies simply on exchanging one amino acid for another in the 3D structure of a fully folded protein but is unable to take account of how these substitutions might affect the process of folding itself. As such, it may often under-estimate the biological effects of missense substitutions, particularly when they affect the elements of underlying secondary structure which drive the process of protein folding *in vivo*. Proline substitutions are well-known for such effects. Proline is often found at the start of α helices, where, due to the rotational constraint imposed by the cyclic nature of the amino acid, it acts to separate regions of differing secondary structure. This property is likely to be particularly important for insertion of multi-pass membrane proteins, where the translocation complex must be able to correctly recognize start-transport and stop-transport signals at each end of the TM helices. In the case of Pro133, this residue separates the flexible helix 3–helix 4 loop from TM helix 4; substitution of proline by leucine will reduce the structural constraint at this boundary, potentially resulting in altered secondary structure leading to misfolding and/or incorrect membrane insertion of the TM helices. Conversely, Thr65 occurs in the flexible loop between helices 1 and 2, where this flexibility is likely to be important for folding of the loop back on itself during membrane insertion to allow correct antiparallel insertion of helices 1 and 2. Due to its cyclic nature, proline substitutions reduce flexibility and increase rigidity in proteins, again potentially leading to aberrant protein folding and/or membrane insertion in this region. It is possible therefore that both the p.(Pro133Leu) and p.(Thr65Pro) substitutions have deleterious effects on protein folding that are not apparent from comparative modelling alone, and thus may have greater biological effects than are evident from such modelling. In support of this hypothesis, analysis of native and variant SLC35D1 sequences using a range of freely available web-based tools indicated that both variants were predicted to cause small but calculable changes to the predicted secondary structure around the variant, including altered stability and position of TM helices, as well as changes to the hydrophobicity at the N-terminal of TM helix 4 (data not shown).

### In vitro substrate specificity of SLC35D1

**Figure 4:**
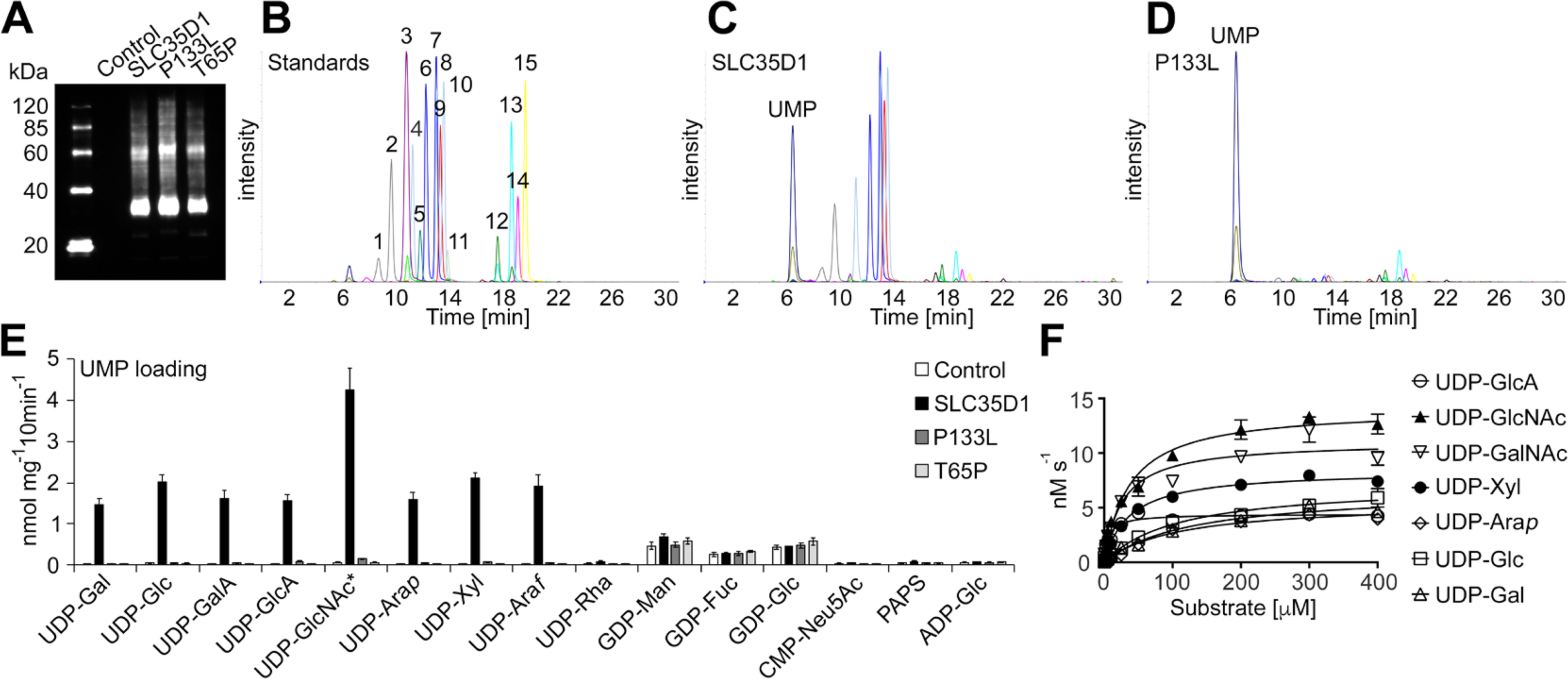
Substrate specificity and kinetics of SLC35D1 and variants. (A) Immunoblot analysis of proteoliposomes including the empty vector (Control), SLC35D1, SLC35D1 p.(Pro133Leu) (P133L) and SLC35D1 p.(Thr65Pro) (T65P). (B) Separation and detection of nucleotide sugar standards (20 pmol each) by multiple reaction monitoring. (C) Representative MRM analysis of SLC35D1-containing proteoliposomes preloaded with 30 mM UMP after conducting the transport assay with 16 nucleotide sugar substrates. (D) Representative MRM analysis of SLC35D1 p.(Pro133Leu)-containing proteoliposomes (P133L) preloaded with 30 mM UMP after conducting the transport assay with 16 nucleotide sugar substrates. (E) Quantification of nucleotide sugar transport into proteoliposomes with Control, SLC35D1, P133L or T65P preloaded with 30 mM UMP after incubation with 16 nucleotide sugar substrates. Data represent the mean and SD of *n* = 4 assays. ^*^ indicates that both UDP-GlcNAc and UDP-GalNAc were employed, reflecting 2-fold increase in signal compared to other substrates. (F) SLS35D1-containing proteoliposomes were preloaded with 30 mM UMP and incubated with the listed substrates at varying concentration (0.5 to 400 μM) for 2 min at 37°C. Data are the mean and SEM of *n* = 4 assays.

SLC35D1 has previously been characterised as an ER localised UDP-GlcA/UDP-GalNAc transporter involved in chondroitin biosynthesis (4). However, previous data indicated that SLC35D1 may also be capable of transporting other nucleotide sugar substrates (7). Thus, to compare accurately functional constraints in the SLC35D1 p.(Pro133Leu) variant, we initially sought to determine the in vitro substrate specificities for SLC35D1 using a recently developed proteoliposome-based transport assay (18). Consequently, SLC35D1 was heterologously expressed in yeast (Fig. 4A) and its ability to transport nucleotide sugars with UMP as counter exchange substrate under competitive conditions was assessed (Fig. 4B; 4C). Our results confirm previous work, indicating that SLC35D1 transports both UDP-GalNAc and UDP-GlcA. However, adding to earlier observations (7), we have observed that SLC35D1 appears to function as a general UDP-sugar transporter in vitro, with the ability to transport UDP-GlcNAc, UDP-GalNAc, UDP-Gal, UDP-Glc, UDP-GalA, UDP-GlcA, UDP-Ara*p*, UDP-Xyl and UDP-Ara*f* (Fig. 4E). Minor transport of GDP-sugars were also observed in the SLC35D1 assay; however, this is likely the result of endogenous yeast transport activity, since GDP-sugar uptake was comparable to that of the corresponding control (Fig. 4E). Notably, compared with the control, SLC35D1 did not have any significant nucleotide sugar transport capability when proteoliposomes were preloaded with AMP, GMP or CMP, indicating that its transport abilities are strictly dependent on the presence of UMP as exchange substrate (Fig. S2).

### Kinetics for multiple substrates for SLC35D1

A detailed analysis of the kinetic parameters for SLC35D1 revealed a *K*_m_ of 6 μM for UDP-GlcA, while the *K*_m_ values for UDP-GlcNAc, UDP-GalNAc and UDP-Xyl were in the range of 27 to 37 μM, those for UDP-Ara*p*, UDP-Glc and UDP-Gal were in the range of 106 to 126 μM (Table 2). To estimate whether the *K*_m_ values for SLC35D1 were within the physiological range of cellular nucleotide sugar concentrations, we quantified the nucleotide sugar pools from different organs harvested from mouse including brain, heart, liver and lungs. Since the identity between human and mouse SLC35D1 protein is about 97%, we concluded that such a comparison between the two species is reasonable. The UDP-sugar contents in the mouse organs range from 1 to 347 pmol·mg^−1^ of fresh weight, respectively (Table 3) and apart from UDP-Ara*p*, the estimated *K*_m_ values for UDP-sugars transported by SLC35D1 are likely to be within the expected physiological range.

**Table 2:** Kinetic parameters of SLC35D1 mediated transport into proteoliposomes

**SLC35D1**	**UDP-GlcA**	**UDP-GlcNAc**	**UDP-GalNAc**	**UDP-Xyl**	**UDP-Ara*p***	**UDP-Glc**	**UDP-Gal**
*K*_m_ [μM]	6 ± 1	37 ± 6	27 ± 5	36 ± 3	106 ± 17	108 ± 14	126 ± 19±
*V*_max_ [nM s^−1^]	4 ± 0	14 ± 1	11 ± 1	8 ± 0	6 ± 0	7 ± 0±	7 ± 0
*k*_cat_ [s^−1^]	0.1	0.4	0.3	0.2	0.1	0.2	0.2

Data points with varying substrate concentrations (0.5 to 400 μM) were acquired. Total replicates were *n* = 4 ± Std. Error. The abundance of SLC35D1 in proteoliposomes was estimated using MRM as outlined in supplementary table 1.

**Table 3:** Nucleotide sugar pools from various mouse organs

**compound**	**brain**	**heart**	**liver**	**lung**
UDP-α-d-Glc	38.5 ± 1.7	13.1 ± 1.7	141.4 ± 4.4	28.1 ± 0.9
UDP-α-d-Gal	16.7 ± 2.1	11.2 ± 2.3	79.0 ± 4.2	12.9 ± 0.2
UDP-α-d-GlcA	6.1 ± 0.3	8.3 ± 1.4	10.1 ± 0.7	10.8 ± 0.2
UDP-α-d-Xyl	1.7 ± 0.1	1.2 ± 0.2	9.1 ± 0.4	1.3 ± 0.1
UDP-β-l-Ara*p*	0.2 ± 0.0	0.03 ± 0.0	0.9 ± 0.1	0.1 ± 0.0
UDP-α-d-GlcNAc/GalNAc	78.7 ± 4.3	62.3 ± 5.3	346.6 ± 10.1	77.0 ± 3.5
GDP-β-l-Fuc	6.1 ± 0.4	3.8 ± 0.5	2.3 ± 0.1	5.5 ± 0.3
GDP-α-d-Man	18.2 ± 1.6	8.3 ± 1.4	18.2 ± 1.1	10.6 ± 0.4
CMP-Neu5Ac	52.9 ± 1.7	27.8 ± 4.3	6.9 ± 0.6	33.4 ± 1.2
PAPS	0.1 ± 0.0	0.1 ± 0.0	1.3 ± 0.1	0.9 ± 0.0

Values are reported as pmol mg^−1^ fresh weight and represent the average of *n* = 3 technical replicates ± STDEV.

### Biochemical characterisation of the SLC35D1 p.(Pro133Leu) variant

To determine whether the missense change in SLC35D1 c.398C > T p.(Pro133Leu) indeed affects SLC35D1 function, we again employed the proteoliposome assay to evaluate its transport capacity (Fig. 4D, P133L). As a control, we also generated and tested the previously characterised missense mutation SLC35D1 c.193A > C p.(Thr65Pro) that results in Schneckenbecken dysplasia (4). The SLC35D1 p.(Thr65Pro) mutant (T65P) has been reported to possess severely diminished activity indicating that this amino acid is critical for the function of SLC35D1 (9). Our data reveal that both mutations significantly affect SLC35D1 function (Fig. 4E); however, while the T65P mutation results in a complete loss-of transport activity, this newly reported P133L variant leads to a dramatic decrease in transport activity, which we estimated to be around 2% to 4% activity compared to that of the SLC35D1 protein.

## Discussion

We report the identification, modelling and characterisation of a new hypomorphic allele of SLC35D1 that results in a p.(Pro133Leu) change that dramatically affects the *in vitro* function of this protein. The identified mutation causes a milder radiological phenotype which we have termed ‘Schneckenbecken-like dysplasia’; of note are the absence of the ‘snail-like’ pelvis that gives the condition its name and the unusual oval ossification of the proximal inferior pubic rami not seen in classic Schneckenbecken dysplasia. While traditional radiology did not suggest the diagnosis, ‘reverse radiology’ following parental whole-exome sequencing provided support to pursue functional studies of SLC35D1 involving modelling and biochemical characterisation.

Functional analysis of the p.(Pro133Leu) variant showed that, like the previously reported p.(Thr133Pro) variant, there was a profound loss of transport activity as a result of the substitution. However, whereas the p.(Thr133Pro) variant displayed complete inactivation of UDP-sugar transport, as previously described (9), the p.(Pro133Leu) variant retained low residual activity of 2 to 4% compared to wildtype. Interestingly, this low but detectable activity is consistent with the milder phenotype and absence of the classical pelvic features observed in our patient compared to the carrier of the p.(Thr65Pro) variant. *In silico* analysis of both variants by comparative protein modelling indicated relatively modest effects on protein structure; however, in both cases the involvement of proline residues and the structural context of each variant suggested that these substitutions could result in misfolding, leading to a more profound effect on biological activity than was apparent from modelling alone. In the context of our functional data, this seems the more likely possibility, and contrasts with the more subtle effects observed for substitution of a number of substrate-binding residues within either the nucleobase- or sugar-binding pockets of Vrg4 (17) or the maize CMP-sialic acid transporter (19). We conclude therefore that both the p.(Pro133Leu) and p.(Thr65Pro) variants are likely to cause a general or global loss of SLC35D1 function as a result of misfolding and/or aberrant membrane insertion, although confirmation would require further histochemical study of the levels and distribution of SLC35D1 variants within cells. Our data nevertheless highlight the importance of using careful functional analysis where possible to test assumptions based on sequence and/or structural data.

The biochemical characterisation of SLC35D1 and variants was undertaken using a multi-substrate transport assay that has been previously used to characterise NSTs from plants and fungi (18, 20–26). Here, we have demonstrated that SLC35D1 appears to be a general UDP-sugar transporter rather than specific for UDP-GlcNAc, UDP-GalNAc and UDP-GlcA as previously reported (4, 7, 9). Under competitive conditions, SLC35D1 was capable of transporting UDP-GlcNAc, UDP-GalNAc, UDP-Gal, UDP-Glc, UDP-GlcA, UDP-GalA, UDP-Ara*p*, UDP-Xyl and UDP-Ara*f*. Moreover, we have been able to determine an apparent *K*_m_ for these substrates that correlates with estimated cellular concentrations. Significantly, SLC35D1 was unable to transport the plant specific nucleotide sugar UDP-Rha and was incapable of transporting UDP-sugars when not employing UMP as a counter ion. These observations indicate that SLC35D1 exhibits distinct substrate specificity. The identification of UDP-Ara*p* in mouse tissue samples is surprising given that, to the best of our knowledge, there are no reports of glycans containing Ara moieties in mammals. However, the presence of UDP-Ara*p* has previously been reported in Chinese hamster ovary (CHO) cells (27).

Overall, these data support a role for SLC35D1 as a general UDP-sugar transporter of humans in providing a biochemical route for nucleotide sugars to the ER lumen for various glycosylation reactions. Human SLC35D1 has previously been characterised as an ER localised UDP-GlcA, UDP-GlcNAc and UDP-GalNAc transporter (7) with a functional role in chondroitin sulfate biosynthesis (4). However, the multi-substrate *in vitro* transport capabilities of SCL35D1 described here in conjunction with phylogenetic clustering (Fig. S3) with ‘general’ UDP-sugar transporters from *Drosophila melanogaster* (Fringe Connection, FRC) (28) and *Caenorhabditis elegans* (Squashed Vulva-7, SQV-7) (29), support its expanded role as a transporter with a broad substrate specificity.

The functional reclassification of SLC35D1 as a general UDP-sugar transporter of the ER confers a role beyond providing substrates for the elongation of chondroitin sulfate chains as previously reported (4). In fact, the disruption of ER-Golgi movement with brefeldin A has previously indicated that chondroitin sulfate synthesising enzymes are localised to the trans-Golgi network (30). Proteoglycans, such as chondroitin sulfate, comprise large GAG chains and are important components of the animal extracellular matrix and cell surface (31). GAG elongation is generally considered to occur in the Golgi apparatus (32, 33) as are the major steps in the biosynthesis of mucin-type *O*-glycans, *N*-linked glycans and glycolipids (34). So what role is there for a general UDP-sugar transporter in the ER? Prior to the generation of GAGs chains within the Golgi apparatus, a tetrasaccharide is added to the core protein to form the base of all GAG chains. The sequential generation of this tetrasaccharide linker (GlcA-Gal-Gal-Xyl-*O*-Ser) appears to be initiated within the ER (33). Further examples of *O*-linked glycosylation within the ER are those that occur on epidermal growth factor (EGF)-like repeats (35). EGF repeats are found in a variety of proteins including growth factors, receptors and many other proteins present in the extracellular matrix (35). EGF repeat containing proteins can contain Xyl and Glc disaccharides (Xyl-Glc-*O*-Ser) and trisaccharides (Xyl-Xyl-Glc-*O*-Ser), single GlcNAc attached to Ser/Thr residues, as well as disaccharide, trisaccharide or tetrasaccharide sequentially extending from an *O*-Fuc residues (Sialic Acid-Gal-GlcNAc-Fuc-*O*-Ser/Thr). Thus, SLC35D1 is likely responsible for the delivery of UDP-Xyl, UDP-Gal, UDP-Glc, UDP-GlcNAc and UDP-GlcA to the ER for the generation of important di-, tri- and tetrasaccharide extensions on proteins prior to further maturation processes within the endomembrane and may also provide substrates for early generation of polysaccharide structures such as GAG chains.

The extension of SLC35D1 substrate specificity to UDP-Xyl and UDP-Glc, both of which are required for the initial *O*-linked glycosylation steps in the GAG tetrasaccharide linker and di- and trisaccharide found on EGF repeat containing proteins, such as the Notch receptor, elevates the functional significance of this transporter beyond its role in the polymerisation of chondroitin sulfate. The requirement for UDP-Xyl in the ER creates a metabolic portioning quandary since UDP-Xyl is biosynthesised by the Golgi-localised UDP-Xyl Synthase (UXS) from UDP-GlcA (36). Thus, it needs to be remobilised to the ER for the above xylosylation reactions. A Golgi localised UDP-Xyl transporter from humans (SLC35B4) has been described (37) with the authors speculating that SLC35B4 is likely involved in UDP-Xyl efflux from the Golgi to the cytosol. Since SLC35D1 displays clear activity towards UDP-Xyl, this transporter provides a mechanism for Golgi synthesised UDP-Xyl to be transported into the ER for essential xylosylation reactions. While UDP-Glc is presumably also required for the initial glycosylation of EGF repeat containing proteins within the ER, this substrate could also be used by UDP-glucose:glycoprotein glucosyltransferase for reglucosylation of malfolded proteins, resulting in their return to the calnexin/calreticulin cycle for refolding (38). Such pleiotropic roles have already been described for homologous UDP-sugar transporters, including FRC from *D. melanogaster* where mutations in *frc* affect both Notch signaling and heparan sulfate biosynthesis (39).

The finding that SLC35D1 is likely a general UDP-sugar transporter located in the ER of humans further supports the critical role of this NST in developmental biology and may better explain the complex phenotype associated with Schneckenbecken dysplasia (4) and ‘Schneckenbecken-like dysplasia’ described here.

## Materials and Methods

### Substrates

Substrates were obtained from the following sources: UDP-α-d-xylose, UDP-β-l-arabinopyranose and UDP-α-d-galacturonic acid (Carbosource Services, Complex Carbohydrate Research Center, Athens, GA); UDP-α-d-glucuronic acid, UDP-α-d-glucose, UDP-α-d-galactose, UDP-*N*-acetyl-α-d-glucosamine, UDP-*N*-acetyl-α-d-galactosamine, GDP-α-d-mannose, GDP-β-l-fucose, GDP-α-d-glucose, adenosine 3′-phosphate 5′ phosphosulfate, CMP-*N*-acetylneuraminic acid and ADP-α-d-glucose (Sigma-Aldrich, St Louis, MO); and UDP-β-l-arabinofuranose (Peptides International, Louisville, KY). UDP-β-l-rhamnose was enzymatically synthesised as described earlier (18).

### Parental exome analysis

Exome library preparation, sequencing, variant annotation and filtering for the couple was undertaken as described previously (40). Variant confirmation and co-segregation was undertaken by PCR and Sanger sequencing (primers available on request). The *in silico* tools Align GVGD, SIFT and PolyPhen were accessed through Alamut Visual (Interactive Biosoftware).

### Protein modelling

Comparative protein modelling of SLC35D1 was carried out using the Swiss-Model web server (16); *in silico* mutagenesis was carried out using the FoldX modelling suite (41).

### Cloning procedures and mutagenesis

Human ORFeome v8.1 SLC35D1 clone (accession BC093786, clone ID ccsbBroadEn_02726) without native stop codon was obtained from the Ultimate ORF clone collection (Thermo Fisher Scientific). For heterologous expression in *Saccharomyces cerevisiae* (yeast) the coding sequence was introduced into the yeast expression vector pYES-DEST52 (Thermo Fisher Scientific) using LR Clonase II (Thermo Fisher Scientific). The c.193A > C (T65P) mutation was introduced by PCR using Phusion polymerase (New England BioLabs) with the following primers: SLC35D1_193A > C_fwd: 5′-AGAGCGTGCTCCCCAATTACAGAT-3′ and SLC35D1_193A > C_rev: 5′-ATCTGTAATTGGGGAGCACGCTCT-3′. The c.398C > T (P133L) mutation was introduced using primers SLC35D1_398C > T_fwd: 5′-CAAAGAAACTGAACTTGCTAATGTTTACAGTTCTGAG-3′, and SLC35D1_398C > T_rev: 5′-CTCAGAACTGTAAACATTAGCAAGTTCAGTTTCTTTG-3′. The resulting PCR products were digested with Dpn1 and transformed into TOP10 *Escherichia coli* (Thermo Fisher Scientific). All mutations were verified by Sanger sequencing.

### Transporter assay

Heterologous expression in *S. cerevisiae* (strain INVSc1), reconstitution of microsomal proteins and subsequent transport activity assays were carried out as previously described (25). Kinetic parameters were calculated by non-linear regression using the Prism7 application (GraphPad Software). Absolute quantification of expressed SLC35D1 in yeast proteoliposomes was undertaken by MRM analysis of a common tryptic peptide to the V5-tag region using a 6460 Triple Quad LC/MS system (Agilent Technologies) as previously described (25) and detailed in Supplementary Table 1. Polyacrylamide gel electrophoreses and immunoblot analyses were conducted as previously described (25) using 2.5 μg of protein derived from liposome preparations and probed using an anti-V5 antibody (Thermo Fisher Scientific).

### Nucleotide sugar extraction from mouse tissue

Mouse tissues (livers, lungs, brains, hearts) were harvested from two individuals (CD-1) and pooled. Tissues were ground to a fine powder in liquid nitrogen using a mortar and pestle. Nucleotide sugars were extracted, purified and quantified by LC-MS/MS as previously described (25).

## Supplementary Material

SLC35D1_Supp_Data_ddz200

## References

[1] Borochowitz Z., Jones K.L., Silbey R., Adomian G., Lachman R. and Rimoin D.L. (1986) A distinct lethal neonatal chondrodysplasia with snail-like pelvis: Schneckenbecken dysplasia. *Am. J. Med. Genet.*, 25, 47–59. 3799723 10.1002/ajmg.1320250107

[2] Camera G., Scarano G., Tronci A., La Cava G. and Mastroiacovo P. (1991) ‘Snail-like pelvis’ chondrodysplasia: a further case report. *Am. J. Med. Genet.*, 40, 513–514. 1746621 10.1002/ajmg.1320400429

[3] Nikkels P.G., Stigter R.H., Knol I.E. and van der Harten H.J. (2001) Schneckenbecken dysplasia, radiology, and histology. *Pediatr. Radiol.*, 31, 27–30. 11200994 10.1007/s002470000357

[4] Hiraoka S., Furuichi T., Nishimura G., Shibata S., Yanagishita M., Rimoin D.L., Superti-Furga A., Nikkels P.G., Ogawa M., Katsuyama K. et al. (2007) Nucleotide-sugar transporter SLC35D1 is critical to chondroitin sulfate synthesis in cartilage and skeletal development in mouse and human. *Nat. Med.*, 13, 1363–1367. 17952091 10.1038/nm1655

[5] Lee H., Nevarez L., Lachman R.S., Wilcox W.R., Krakow D., Cohn D.H. and University of Washington Center for Mendelian Genomics (2015) A second locus for schneckenbecken dysplasia identified by a mutation in the gene encoding inositol polyphosphate phosphatase-like 1 (INPPL1). *Am. J. Med. Genet. A*, 167, 2470–2473. 10.1002/ajmg.a.37173 PMC5036935 25997753

[6] Song Z.W. (2013) Roles of the nucleotide sugar transporters (SLC35 family) in health and disease. *Mol. Aspects Med.*, 34, 590–600. 23506892 10.1016/j.mam.2012.12.004

[7] Muraoka M., Kawakita M. and Ishida N. (2001) Molecular characterization of human UDP-glucuronic acid/UDP-*N*-acetylgalactosamine transporter, a novel nucleotide sugar transporter with dual substrate specificity. *FEBS Lett.*, 495, 87–93. 11322953 10.1016/s0014-5793(01)02358-4

[8] Sugahara K. and Kitagawa H. (2000) Recent advances in the study of the biosynthesis and functions of sulfated glycosaminoglycans. *Curr. Opin. Struct. Biol.*, 10, 518–527. 11042448 10.1016/s0959-440x(00)00125-1

[9] Furuichi T., Kayserili H., Hiraoka S., Nishimura G., Ohashi H., Alanay Y., Lerena J.C., Aslanger A.D., Koseki H., Cohn D.H. et al. (2009) Identification of loss-of-function mutations of SLC35D1 in patients with Schneckenbecken dysplasia, but not with other severe spondylodysplastic dysplasias group diseases. *J. Med. Genet.*, 46, 562–568. 19508970 10.1136/jmg.2008.065201 PMC4144354

[10] Lek M., Karczewski K.J., Minikel E.V., Samocha K.E., Banks E., Fennell T., O’Donnell-Luria A.H., Ware J.S., Hill A.J., Cummings B.B. et al. (2016) Analysis of protein-coding genetic variation in 60,706 humans. *Nature*, 536, 285–291. 27535533 10.1038/nature19057 PMC5018207

[11] Mathe E., Olivier M., Kato S., Ishioka C., Hainaut P. and Tavtigian S.V. (2006) Computational approaches for predicting the biological effect of p53 missense mutations: a comparison of three sequence analysis based methods. *Nucleic Acids Res.*, 34, 1317–1325. 16522644 10.1093/nar/gkj518 PMC1390679

[12] Tavtigian S.V., Deffenbaugh A.M., Yin L., Judkins T., Scholl T., Samollow P.B., de Silva D., Zharkikh A. and Thomas A. (2006) Comprehensive statistical study of 452 *BRCA1* missense substitutions with classification of eight recurrent substitutions as neutral. *J. Med. Genet.*, 43, 295–305. 16014699 10.1136/jmg.2005.033878 PMC2563222

[13] Adzhubei I.A., Schmidt S., Peshkin L., Ramensky V.E., Gerasimova A., Bork P., Kondrashov A.S. and Sunyaev S.R. (2010) A method and server for predicting damaging missense mutations. *Nat. Methods*, 7, 248–249. 20354512 10.1038/nmeth0410-248 PMC2855889

[14] Tavtigian S.V., Byrnes G.B., Goldgar D.E. and Thomas A. (2008) Classification of rare missense substitutions, using risk surfaces, with genetic- and molecular-epidemiology applications. *Hum. Mutat.*, 29, 1342–1354. 18951461 10.1002/humu.20896 PMC3938023

[15] Tavtigian S.V., Greenblatt M.S., Lesueur F., Byrnes G.B. and IARC Unclassified Genetic Variants Working Group (2008) *In silico* analysis of missense substitutions using sequence-alignment based methods. *Hum. Mutat.*, 29, 1327–1336. 18951440 10.1002/humu.20892 PMC3431198

[16] Biasini M., Bienert S., Waterhouse A., Arnold K., Studer G., Schmidt T., Kiefer F., Gallo Cassarino T., Bertoni M., Bordoli L. et al. (2014) SWISS-MODEL: modelling protein tertiary and quaternary structure using evolutionary information. *Nucleic Acids Res.*, 42, W252–W258. 24782522 10.1093/nar/gku340 PMC4086089

[17] Parker J.L. and Newstead S. (2017) Structural basis of nucleotide sugar transport across the Golgi membrane. *Nature*, 551, 521–524. 29143814 10.1038/nature24464 PMC5701743

[18] Rautengarten C., Ebert B., Moreno I., Temple H., Herter T., Link B., Donas-Cofre D., Moreno A., Saez-Aguayo S., Blanco F. et al. (2014) The Golgi localized bifunctional UDP-rhamnose/UDP-galactose transporter family of Arabidopsis. *Proc. Natl. Acad. Sci. U. S. A.*, 111, 11563–11568. 25053812 10.1073/pnas.1406073111 PMC4128141

[19] Nji E., Gulati A., Qureshi A.A., Coincon M. and Drew D. (2019) Structural basis for the delivery of activated sialic acid into Golgi for sialyation. *Nat. Struct. Mol. Biol.*, 26, 415–423. 31133698 10.1038/s41594-019-0225-y

[20] Ebert B., Rautengarten C., Guo X., Xiong G., Stonebloom S., Smith-Moritz A.M., Herter T., Chan L.J., Adams P.D., Petzold C.J. et al. (2015) Identification and characterization of a Golgi-localized UDP-xylose transporter family from Arabidopsis. *Plant Cell*, 27, 1218–1227. 25804536 10.1105/tpc.114.133827 PMC4558686

[21] Ebert B., Rautengarten C., McFarlane H.E., Rupasinghe T., Zeng W., Ford K., Scheller H.V., Bacic A., Roessner U., Persson S. et al. (2018) A Golgi UDP-GlcNAc transporter delivers substrates for N-linked glycans and sphingolipids. *Nature Plants*, 4, 792–801. 30224661 10.1038/s41477-018-0235-5

[22] Li L.X., Rautengarten C., Heazlewood J.L. and Doering T.L. (2018) UDP-glucuronic acid transport is required for virulence of *Cryptococcus neoformans*. *MBio*, 9, e02319–17. 29382737 10.1128/mBio.02319-17 PMC5790919

[23] Li L.X., Rautengarten C., Heazlewood J.L. and Doering T.L. (2018) Xylose donor transport is critical for fungal virulence. *PLoS Pathog.*, 14, e1006765. 29346417 10.1371/journal.ppat.1006765 PMC5773217

[24] Rautengarten C., Birdseye D., Pattathil S., McFarlane H.E., Saez-Aguayo S., Orellana A., Persson S., Hahn M.G., Scheller H.V., Heazlewood J.L. et al. (2017) The elaborate route for UDP-arabinose delivery into the Golgi of plants. *Proc. Natl. Acad. Sci. U. S. A.*, 114, 4261–4266. 28373556 10.1073/pnas.1701894114 PMC5402404

[25] Rautengarten C., Ebert B., Liu L., Stonebloom S., Smith-Moritz A.M., Pauly M., Orellana A., Scheller H.V. and Heazlewood J.L. (2016) The Arabidopsis Golgi-localized GDP-L-fucose transporter is required for plant development. *Nat. Commun.*, 7, 12119. 27381418 10.1038/ncomms12119 PMC4935801

[26] Saez-Aguayo S., Rautengarten C., Temple H., Sanhueza D., Ejsmentewicz T., Sandoval-Ibanez O., Donas D., Parra-Rojas J.P., Ebert B., Lehner A. et al. (2017) UUAT1 is a Golgi-localized UDP-Uronic acid transporter that modulates the polysaccharide composition of Arabidopsis seed mucilage. *Plant Cell*, 29, 129–143. 28062750 10.1105/tpc.16.00465 PMC5304346

[27] Pabst M., Grass J., Fischl R., Leonard R., Jin C., Hinterkorner G., Borth N. and Altmann F. (2010) Nucleotide and nucleotide sugar analysis by liquid chromatography-electrospray ionization-mass spectrometry on surface-conditioned porous graphitic carbon. *Anal. Chem.*, 82, 9782–9788. 21043458 10.1021/ac101975k PMC2995335

[28] Goto S., Taniguchi M., Muraoka M., Toyoda H., Sado Y., Kawakita M. and Hayashi S. (2001) UDP-sugar transporter implicated in glycosylation and processing of Notch. *Nat. Cell Biol.*, 3, 816–822. 11533661 10.1038/ncb0901-816

[29] Berninsone P., Hwang H.Y., Zemtseva I., Horvitz H.R. and Hirschberg C.B. (2001) SQV-7, a protein involved in *Caenorhabditis elegans* epithelial invagination and early embryogenesis, transports UDP-glucuronic acid, UDP-*N*-acetylgalactosamine, and UDP-galactose. *Proc. Natl. Acad. Sci. U. S. A.*, 98, 3738–3743. 11259660 10.1073/pnas.061593098 PMC31122

[30] Spiro R.C., Freeze H.H., Sampath D. and Garcia J.A. (1991) Uncoupling of chondroitin sulfate glycosaminoglycan synthesis by brefeldin A. *J. Cell Biol.*, 115, 1463–1473. 1955486 10.1083/jcb.115.5.1463 PMC2289244

[31] Pomin V.H. and Mulloy B. (2018) Glycosaminoglycans and proteoglycans. *Pharmaceuticals*, 11, 27. 10.3390/ph11010027 PMC5874723 29495527

[32] Uyama T., Kitagawa H. and Sugahara K. (2007) In Kamerling H. (ed), *Comprehensive Glycoscience*. Elsevier, Oxford, pp. 79–104.

[33] Prydz K. (2015) Determinants of glycosaminoglycan (GAG) structure. *Biomolecules*, 5, 2003. 26308067 10.3390/biom5032003 PMC4598785

[34] Stanley P. (2011) Golgi glycosylation. *Cold Spring Harb. Perspect. Biol.*, 3, a005199. 21441588 10.1101/cshperspect.a005199 PMC3062213

[35] Haltom A.R. and Jafar-Nejad H. (2015) The multiple roles of epidermal growth factor repeat *O*-glycans in animal development. *Glycobiology*, 25, 1027–1042. 26175457 10.1093/glycob/cwv052 PMC4551148

[36] Moriarity J.L., Hurt K.J., Resnick A.C., Storm P.B., Laroy W., Schnaar R.L. and Snyder S.H. (2002) UDP-glucuronate decarboxylase, a key enzyme in proteoglycan synthesis—cloning, characterization, and localization. *J. Biol. Chem.*, 277, 16968–16975. 11877387 10.1074/jbc.M109316200

[37] Ashikov A., Routier F., Fuhlrott J., Helmus Y., Wild M., Gerardy-Schahn R. and Bakker H. (2005) The human solute carrier gene SLC35B4 encodes a bifunctional nucleotide sugar transporter with specificity for UDP-xylose and UDP-*N*-acetylglucosamine. *J. Biol. Chem.*, 280, 27230–27235. 15911612 10.1074/jbc.M504783200

[38] Suzuki T., Tanabe K. and Funakoshi Y. (2007) In Kamerling H. (ed), *Comprehensive Glycoscience*. Elsevier, Oxford, pp. 129–149.

[39] Selva E.M., Hong K., Baeg G.H., Beverley S.M., Turco S.J., Perrimon N. and Hacker U. (2001) Dual role of the fringe connection gene in both heparan sulphate and fringe-dependent signalling events. *Nat. Cell Biol.*, 3, 809–815. 11533660 10.1038/ncb0901-809

[40] Stals K.L., Wakeling M., Baptista J., Caswell R., Parrish A., Rankin J., Tysoe C., Jones G., Gunning A.C., Allen H.L. et al. (2018) Diagnosis of lethal or prenatal-onset autosomal recessive disorders by parental exome sequencing. *Prenatal Diagn.*, 38, 33–43. 10.1002/pd.5175 PMC5836855 29096039

[41] Schymkowitz J., Borg J., Stricher F., Nys R., Rousseau F. and Serrano L. (2005) The FoldX web server: an online force field. *Nucleic Acids Res.*, 33, W382–W388. 15980494 10.1093/nar/gki387 PMC1160148

