# Supplementary material for: A hypomorphic allele of SLC35D1 results in Schneckenbecken-like dysplasia
Source: Hum Mol Genet. 2019 Aug 19;28(21):3543–51. doi: 10.1093/hmg/ddz200 (PMC6927460; doi:10.1093/hmg/ddz200)
Supplement: SLC35D1_Supp_Data_ddz200 [file slc35d1_supp_data_ddz200.pdf]

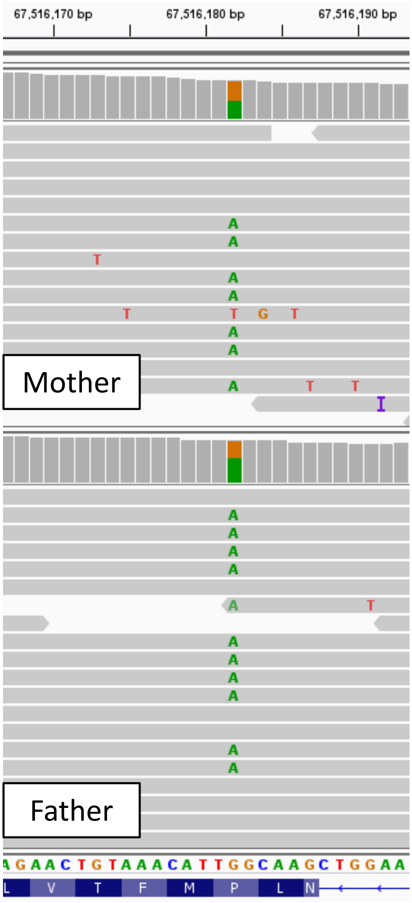

SLC35D1

Figure S1. Parental exome sequence data aligned with the *SLC35D1* region.

Exome sequence data from parents were processed according to previous methods (40). Filtered reads were visualized using the integrated genomics viewer (IGV). The screenshot shows the heterozgyous c.398C>T identified in the mother (top panel) and father (bottom panel).

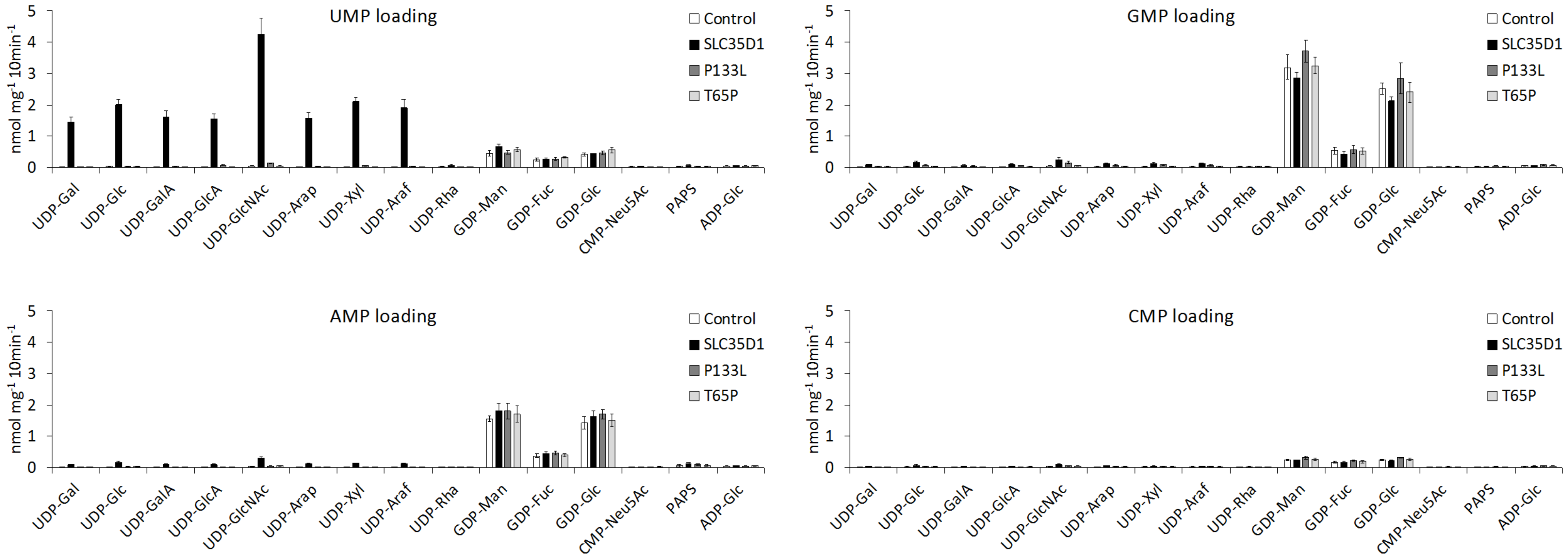

Figure S2. Exchange substrate specificity of SLC35D1.

Quantification of nucleotide sugar transport into proteoliposomes preloaded with UMP (identical to Fig. 4E), GMP, CMP or AMP containing SLC35D1 and the two variants. Data represent the mean and SD of $n = 4$ assays.

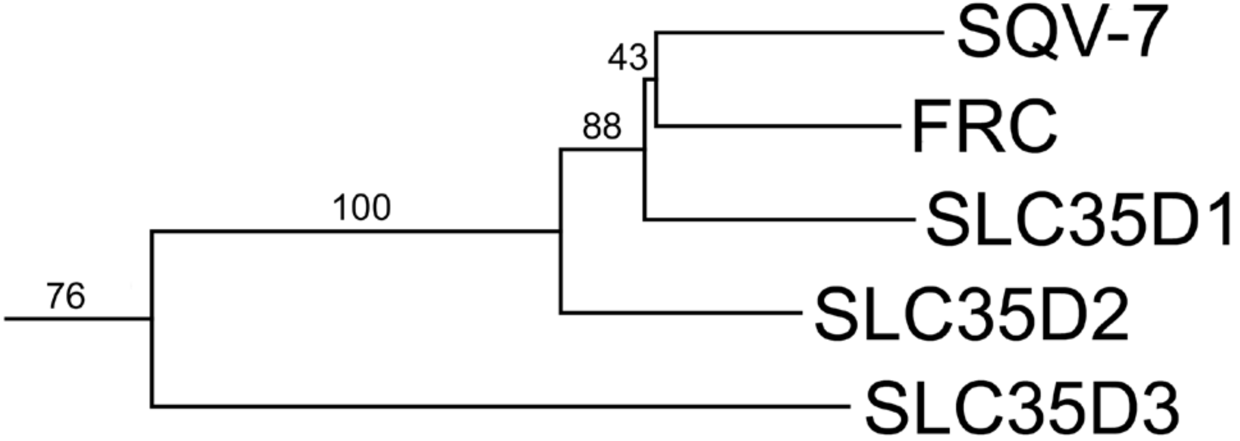

Figure S3. Phylogenetic analysis of the human SLC35D subclade including SLC35D1 with orthologs from *C.elegans* and *Drosophila*.

The full-length amino acid sequences of all human SLC35 family members, *Caenorhabditis elegans* SQV-7, and *Drosophila melanogaster* FRC were aligned with Clustal Omega and the tree was generated using the MEGA7 application. SLC35 subclade D was extracted. Numbers at the nodes indicate bootstrap values calculated for 1000 replicates.

Table S1. Calculations for amount of expressed protein in proteoliposomes used for transport assays.

NST	MM (Da)	fmol (STDEV) (in 10 µg)	ng (STDEV) (in 10 µg)	Total protein (%)
SLC35D1	44113.7	2327.9 ± 104.4	102.7 ± 4.6	1.03 ± 0.05

The molecular mass is the estimated monoisotopic mass including the V5-tag and 6-His tag using the Compute pI/Mw tool at ExPASy (http://web.expasy.org/). The amount (fmol) in the sample was estimated using LC-MS/MS (MRM) quantitation of a shared C-terminal peptide (SRGPFEGKPIPNPLLGLDSTR). Samples are mean (*n*=3) ± STDEV.

Table S2.

**Feature**	**Present Study**	**Furuchi et al., (2009)**				
		**Patient 1**	**Patient 2**	**Patient 3**	**Patient 4**	**Patient 5**
**Clinical**						
Gestational age (weeks)	28	18	22	20	20	28
Ethnicity	Arabian	Caucasian	Turkish	Turkish	Turkish	Brazilian
SLC35D1 mutation	+	+	+	+	-	-
**Radiological**						
Snail-like pelvis	-	+++	+++	+++	+++	+
Ossification of superior pubic rami*	Yes	Yes	Yes	Yes	No	Yes
Unusual oval ossification of proximal inferior pubic rami	Yes	No	No	No	No	No
Bell-shaped thorax	+	+	+	+	+	+
Handle-bar clavicles	+	+	+	+	+	+
Metaphyseal flaring	+	++	++	++	++	++
Angular humerus	-	-	-	-	-	+
Narrow IP* distance	Subtle	+	+	+	+	+
Platyspondyly	-	+	+	+	+	+
Coronal clefts	+	-	-	-	-	-
Ossification of posterior vertebral arches	Normal	Normal	Normal	Normal	Poor	Normal
Sacral ossification	Normal	Relatively normal	Relatively normal	Relatively normal	Poor	Normal

* Normal superior pubic rami excludes a type II collagenopathy, an initial consideration in our case

** IP = interpediculate distance
